# Decomposing socio-economic inequalities in antenatal care utilisation in 12 Southern African Development Community countries

**DOI:** 10.1016/j.ssmph.2021.101004

**Published:** 2021-12-16

**Authors:** Keolebogile M. Selebano, John E. Ataguba

**Affiliations:** a Health Economics Unit, School of Public Health and Family Medicine, Health Sciences Faculty, University of Cape Town, Anzio Road, Observatory, 7925, South Africa; b Sustainable Health Financing Unit, Clinton Health Access Initiative, 1166, Francis Baard St, Hatfield, Pretoria, South Africa

**Keywords:** Antenatal care, Maternal care, Socioeconomic inequality, Southern african development community

## Abstract

Although many countries are making progress towards achieving the global sustainable development goals, sub-Saharan Africa (SSA) lags behind. SSA bears a relatively higher burden of maternal morbidity and mortality than other regions despite existing cost-effective interventions. This paper assesses antenatal care (ANC) service utilisation among women in the Southern African Development Community (SADC) countries, one of the four SSA regions. Specifically, it assesses socioeconomic inequality in the number of ANC visits, use of no ANC service, between one and three ANC visits and at least four ANC visits, previously recommended by the World Health Organization (WHO). Data come from the most recent Demographic and Health Surveys in twelve SADC countries. Wagstaff's normalised concentration index (CI) was used to assess socioeconomic inequalities. Factors explaining these inequalities were assessed using a standard method and similar variables contained in the DHS data. A positive CI means that the variable of interest is concentrated among wealthier women, while a negative CI signified the opposite. The paper found that wealthier women in the SADC countries are generally more likely to have more ANC visits than their poorer counterparts. Apart from Zambia, the CIs were positive for inequalities in at least 4 ANC visits and negative for between 1 and 3 ANC visits. Women from poorer backgrounds significantly report no ANC visits than wealthier women. Apart from the portion that was not explainable due to limitations in the variables included in the model, critical social determinants of health, including wealth, education and the number of children, explain socioeconomic inequalities in ANC coverage in SADC. A vital policy consideration is not to leave any woman behind. Therefore, addressing access barriers and critical social determinants of ANC inequalities, such as women's education and economic well-being, can potentially redress inequalities in ANC coverage in the SADC region.

## Highlights

• Wealthier women have more antenatal care (ANC) visits than their poorer counterparts.
• Women from poorer backgrounds report no ANC visits more often than wealthier women.
• Wealth and education are key drivers of socioeconomic inequalities in ANC coverage.
• Poorer women are being left behind in accessing maternal health services in Africa.

## 1 Introduction

Maternal mortality ratio in sub-Saharan Africa (SSA) reduced by half between 1990 and 2013 (United Nations Development Programme, 2015a). Nevertheless, the burden of maternal mortality remains higher in SSA compared to other regions despite existing cost-effective interventions (Darmstadt et al., 2005; Adam et al., 2005). Beyond the health sector, maternal education, sociocultural practices, good hygiene practices and adequate nutrition are examples of factors of considerable importance in reducing maternal deaths in many developing countries (Najafizada et al., 2017; UNFPA, 2012). Unfortunately, many of these developing countries report poor indicators for essential social determinants of maternal morbidity and mortality (Gil-González et al., 2006; UNFPA, 2012). Although social environment and economic circumstances significantly affect a woman's chances of surviving pregnancy and childbirth, quality antenatal care comes up as a significantly cost-effective intervention both at the individual and population levels (Adam et al., 2005). It is not just about the number of ANC visits per se but the quality of services, including the content and timing of visits, because quality antenatal care plays a significant role in improving and maintaining maternal health (Kanyangarara et al., 2017). A country like Sierra Leone with one of the highest proportions of pregnant women receiving at least four antenatal care visits in SSA (Ataguba, 2018), has one of the highest maternal mortality ratios in Africa, partly because of poor quality antenatal care services and other critical social determinants of maternal morbidity and mortality (Koroma et al., 2017). The global sustainable development goals (SDGs) aim to reduce maternal mortality, but considerable challenges are facing many developing countries, which may limit significant progress in achieving the goal of reducing maternal mortality to less than 70 per 100 000 live births by 2030 (World Health Organization, 2019, United Nations Development Programme, 2015a United Nations Development Programme (2015b)). Although the factors that affect maternal mortality are broader than access to maternal health services, the continuum of care inclusive of the use of antenatal care (ANC) services, skilled birth attendance (SBA) and postnatal care (PNC) services remain beneficial in reducing maternal mortality and improving the health outcomes of mothers and newborns.

With an average of 4.7 children per woman, SSA's fertility rate is the highest compared to other regions in the world (United Nations, 2015). The high fertility rate means that the number of births occurring in SSA will continue to outstrip those in other world regions. Thus, quality maternal health services, including antenatal care, are needed to ensure a healthy pregnancy experience and journey for women in Africa (World Health Organization, 2016). However, within countries, women from poorer households generally access far less maternal care than women from wealthier households (Nwosu & Ataguba, 2019; Silal et al., 2012; Andrade et al., 2012; Sharma et al., 2007). In many cases, ANC service utilisation predicts SBA and the frequency of PNC visits (Silal et al., 2012; Kerber et al., 2007). This paper assesses the use of ANC services, the point of entry into the health system by many pregnant women, in the Southern African Development Community (SADC) countries. Specifically, it assesses socioeconomic inequality in the use of no ANC service, between one and three ANC visits and at least four ANC visits, as previously recommended by the World Health Organization (WHO) before 2016 (World Health Organization, 2016). To note, while the quality of ANC service utilisation remains critical, the WHO revised its recommendations to a minimum of 6 ANC *contacts* for uncomplicated pregnancies as this increases the likelihood of receiving effective maternal health interventions compared to the minimum of 4 ANC visits (World Health Organization, 2016).

## 2 The SADC countries in brief

The SADC countries are a group of 16 countries located within the southern and eastern parts of Africa, with historical and cultural affinities (SADC, 2021). The SADC region's population is over 360 million, and the average life expectancy at birth varies from 54 years in Lesotho to 74 years in Mauritius (World Bank, 2021). Despite the shared goals for a common future for the SADC countries, there still exist differences between these countries as some (e.g. Botswana, Namibia and South Africa) are relatively wealthier than others (e.g., Lesotho and Malawi). Out-of-pocket (OOP) spending on health as a share of current health expenditure, a significant indicator of barriers to health service utilisation, varies by country, with South Africa recording a minimal share (World Health Organization, 2021a). These countries have a history of user fee abolition to improve maternal health service utilisation, with free antenatal care services at public facilities being common (Masiye et al., 2016; Manthalu et al., 2016; Ridde & Morestin, 2011). Table 1 contains some indicators for the SADC countries.

**Table 1:** Selected indicators, SADC countries.

	Percentage of maternal deaths among deaths of female reproductive age (2017)^a^	Maternal mortality ratio (MMR) (2017)^a^	ANC coverage (% with at least 4 visits, varied years)^b^
Angola	14	241	62.1 (2016)
Botswana	4	144	–
Comoros	13	273	57.5 (2012)
Democratic Republic of Congo	23	473	48.3 (2013/14)
Eswatini	6	437	81.7 (2006/07)
Lesotho	6	544	74.9 (2014)
Madagascar	16	335	58.8 (2016)
Malawi	15	349	50.8 (2015/16)
Mauritius	2	61	–
Mozambique	9	289	52.2 (2015)
Namibia	5	195	81.5 (2013)
Seychelles	3	53	–
South Africa	2	119	76.0 (2016)
Tanzania	22	524	50.9 (2015/16)
Zambia	8	213	56.0 (2013/14)
Zimbabwe	9	458	75.9 (2015)

Notes: ^a^World Health Organization (2021b). The WHO defines maternal death as the death of a woman while pregnant or within 42 days of pregnancy termination. The death could result from any pregnancy-related cause or aggravated by the pregnancy or the management of pregnancy but excludes accidental or incidental causes unrelated to the pregnancy.

b Demographic and Health Survey data for various countries.

## 3 Methods

### 3.1 Data

Data come from the latest Demographic and Health Surveys (DHS) for SADC countries with available data (twelve of the sixteen SADC countries) as of October 2021. The Union of Comoros was not included in the analysis because the latest data are for 2012, and it only became a full member of the SADC countries in August 2018 (SADC, 2021). The DHS use standardised questions to collect information mainly from women of reproductive age (i.e. aged between 15 and 49 years) (Rutstein & Rojas, 2006). The DHS datasets are cross-sectional and nationally representative, with information on women's sociodemographic and socioeconomic characteristics and maternal health service utilisation (DHS Program, 2021). Table 2 contains a summary of the DHS datasets for available countries.

**Table 2:** Sample size per SADC country.

Country	Year	Sample size*
Angola	2015/16	14,379
Botswana	N/A	N/A
Comoros^a^	N/A	N/A
Democratic Republic of Congo	2013/14	18,827
Eswatini	2006/07	6,621
Lesotho	2014	17,375
Madagascar	2008/09	24,562
Malawi	2015/16	13,745
Mauritius	N/A	N/A
Mozambique	2011	10,018
Namibia	2013	4,987
Seychelles	N/A	N/A
South Africa	2016	13,266
Tanzania	2015/16	8,514
Zambia	2018	13,683
Zimbabwe	2015	9,955

Notes: * Sample size = number of women aged 15–49 years.

a Comoros only became a full member in 2018, and the latest DHS dataset was for 2012.

### 3.2 Study variables

Three mutually exclusive variables were created to assess socioeconomic inequality in each of the variables critically: 1) No ANC visits (i.e. when a woman with a live birth in the specified period did not have any ANC visit; 0 ANC) 2) At least one but less than four ANC visits (i.e. having between one and three visits; *1–*3 ANC), and 3) At least four ANC visits (i.e. a woman with at least four ANC visits; *≥* 4 ANC or *4+ ANC*). A fourth encompassing category (ANC intensity) uses the total number of ANC visits that a pregnant woman had received.

The DHS does not directly report a household's expenditure or income but contains information on household assets or a wealth index developed based on a method by Rutstein and Johnson (2004). This paper uses the wealth index as a proxy for socioeconomic status (SES). This index was constructed from household asset data, including access to sanitation facilities, type of flooring material and source of drinking water.

### 3.3 Analytical methods

A comparative analysis of ANC utilisation in the twelve SADC countries was done to give a descriptive assessment of inequalities in the use of antenatal care. This analysis uses equity stratifiers such as type of residence, highest education level, respondents’ occupation and wealth quintiles.

### 3.4 Assessing inequality in antenatal care utilisation

Socioeconomic inequality in the distribution of ANC utilisation was assessed using concentration indices (Wagstaff et al., 1991). Two key variables used to estimate the concentration index are ANC utilisation as a health variable of interest (i.e. 0 ANC, *1–*3 ANC, *4+ ANC* or ANC intensity) and SES using the wealth index.

The standard concentration index is estimated as twice the covariance between the ANC utilisation variable ($H_{i}$) and the relative rank of women using the SES measure ($R_{i}$), divided by the mean of the ANC utilisation variable ($\mu_{H}$) (Wagstaff et al., 1991).

(1) $$C_{H}=\frac{2*cov\left(H_{i},R_{i}\right)}{\mu_{H}}$$

This standard concentration index was used to assess socioeconomic inequalities in the number of ANC visits (i.e. ANC intensity). However, because the other key mutually exclusive variables are dichotomous (i.e. 0 ANC, *1–*3 ANC, *4+ ANC*), the standard concentration index will not range from −1 to +1 (Wagstaff, 2005). The standard concentration index in Equation (1) was normalised using the approach proposed by Wagstaff (2005). Generally, a negative valued concentration index (including the normalised index) corresponds to a higher distribution of ANC service utilisation among women from poorer socioeconomic backgrounds. A positive-valued index signifies a higher utilisation distribution among wealthier women (Kakwani et al., 1997). Also, for interpretation, a positive-valued concentration index can be interpreted as “pro-rich” while a negative index value as “pro-poor.”

The concentration index for ANC intensity was decomposed to identify factors that explain observed socioeconomic inequalities in ANC coverage in SADC countries (Wagstaff et al., 2003). Let us define the relationship between ANC intensity ($H_{i}$) and a set of explanatory variables or factors ($z_{ji}$) as:

(2) $$H_{i}=\alpha +\sum_{j}\beta_{j}z_{ji}+\varepsilon_{i}$$

where α and β are ordinary least squares parameter estimates and ε is the error term.

Wagstaff et al. (2003) use the relationship in Equation (2) to decompose the concentration index in Equation (1) ($C_{H}$) into two major components:

(3) $$C_{H}=\underset{Explained}{\underbrace{\sum_{j=1}^{J}\left(\frac{\beta_{j}{\bar{z}}_{j}}{\mu_{H}}\right)C_{j}}}+\underset{Unexplained}{\underbrace{\left(\frac{GC_{\varepsilon }}{\mu_{H}}\right)}}$$

where $C_{j}$ is the *j*-th contributing factor's concentration index, and $\frac{\beta_{j}{\bar{z}}_{j}}{\mu_{H}}$ is the elasticity of ANC intensity to marginal changes in the *j*-th explanatory variable or factor. The generalised concentration index of the error term is denoted by $GC_{\varepsilon }$. The explained component (i.e. $\left(\frac{\beta_{j}{\bar{z}}_{j}}{\mu_{H}}\right)C_{j}$) is factor *j*'s contribution to socioeconomic inequality in ANC intensity. Explanatory variables or factors used in this paper include the woman's age, education, employment, urban or rural location, region of residence, socioeconomic quintiles, and the total number of children for each woman. These variables featured prominently in previous studies (Obse & Ataguba, 2021; Rosário et al., 2019; Nagdeva, 2009; Shibre et al., 2020; Yaya et al., 2016; McTavish et al., 2010). A woman's total number of children was included in the model to capture multigravida and a woman's previous ANC utilisation experiences that may affect current service utilisation. Interpreting the contributions for each factor ($\left(\frac{\beta_{j}{\bar{z}}_{j}}{\mu_{H}}\right)C_{j}$)) is straightforward. With a positive concentration index, for example, a positive contribution of a factor means that the factor contributes to the concentration of inequalities in ANC utilisation among wealthier women. The unexplained component, $\left(\frac{GC_{\varepsilon }}{\mu_{H}}\right)$, is also called the residual and accounts, among other things, for unexplained factors. The value of the unexplained component should be close to zero for a well-specified model that includes all relevant variables. The values of each component, including their associated standard errors, were computed in Stata using a user-developed computer routine (Bilger et al., 2017). Specifically, bootstrap methods are used to obtain standard errors in Equation (3) with 500 replications (Efron, 1987; Efron & Tibshirani, 1986), accounting for the sampling structure of each DHS.

Stata 15 was used to perform all analyses in the paper (StataCorp, 2017).

## 4 Results

**Fig. 1:**
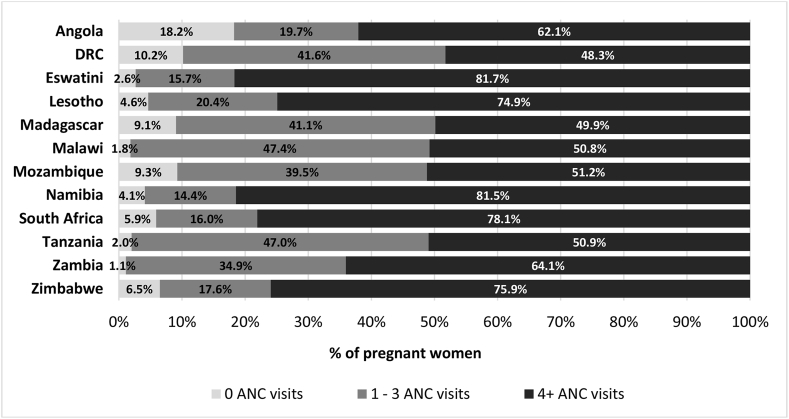
Antenatal care coverage, Southern Africa Development Community. *Note*: DRC = Democratic Republic of Congo.

A substantial proportion of women had at least 4 ANC visits, ranging from 48.3% (Democratic Republic of Congo) to 81.7% (Eswatini). Although over 18% of women had no ANC visits in Angola, the proportion was smaller for other countries (Fig. 1). Also, a sizable proportion of women in the SADC countries recorded between one and three ANC visits, with the highest proportion in Malawi and Tanzania (∼47%) (Fig. 1). More than 70% of pregnant women in Eswatini, Lesotho, Namibia, South Africa and Zimbabwe had at least 4 ANC visits.

**Fig. 2:**
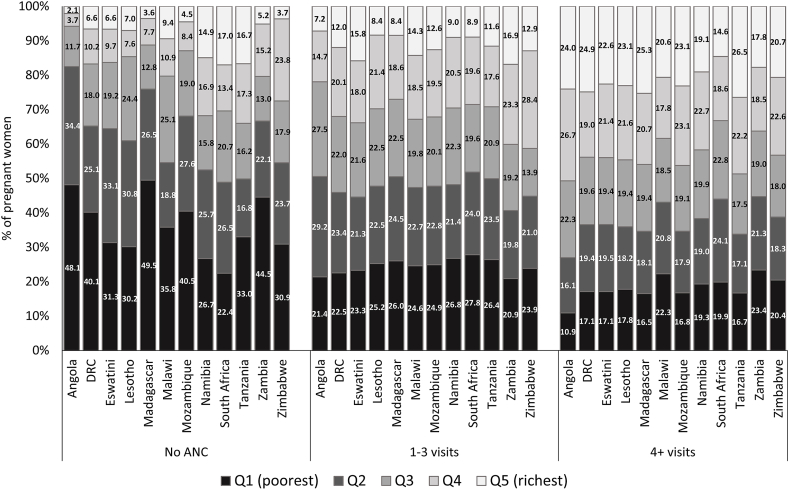
Distribution of ANC utilisation in the Southern Africa Development Community countries by socioeconomic groups. *Note*: DRC = Democratic Republic of Congo.

**Fig. 3:**
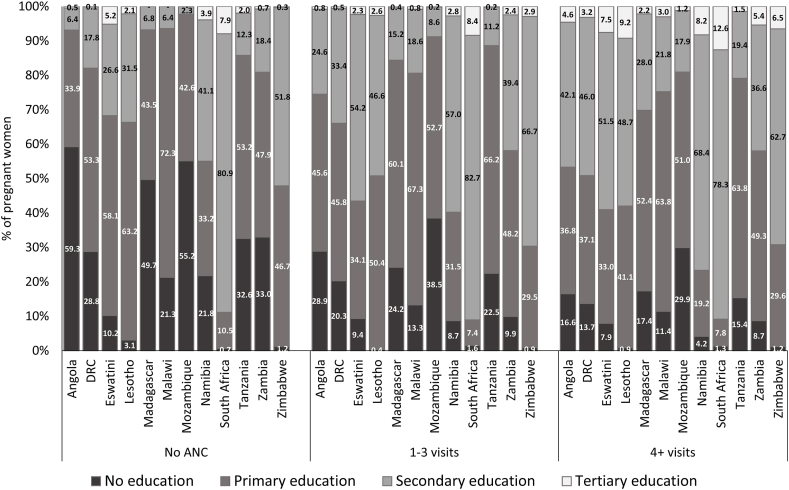
Distribution of ANC utilisation in the Southern Africa Development Community countries by education category. *Note*: DRC = Democratic Republic of Congo.

**Fig. 4:**
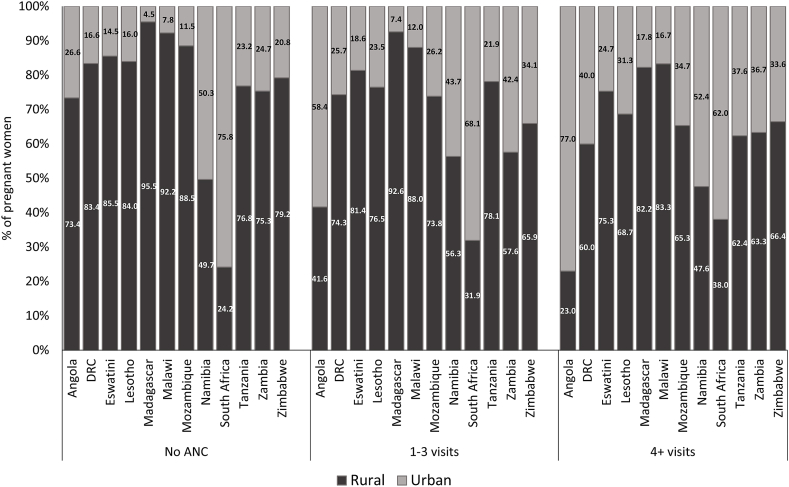
Distribution of ANC utilisation in the Southern Africa Development Community countries by location. *Note*: DRC = Democratic Republic of Congo.

The utilisation of ANC services has a marked socioeconomic gradient (Fig. 2). In all the countries shown in Fig. 2, the proportion of women with no ANC visits, categorised in the poorest quintile, is higher than that for women with at least four visits. For example, in Tanzania, 33.0% of women with no ANC visits are in the poorest quintile compared to 16.7% for women with at least four ANC visits. Apart from South Africa, the proportion of women with at least four ANC visits, categorised in the wealthiest quintile, is greater than the corresponding proportion for women with no ANC visits. The socioeconomic gradient also appears in Fig. 3 for education attainment. The proportion of women with no formal education is higher for women with no ANC visits than for women with at least four ANC visits in most countries. In Angola, for instance, 59.3% of women without any ANC visits had no formal education compared to 16.6% for women with at least four ANC visits. A small proportion of women had attained tertiary education in these countries. Therefore, tertiary education alone did not significantly impact the gradient as primary and secondary education (Fig. 2). Apart from Namibia and South Africa, women in rural locations were more likely not to have ANC visits than their urban counterparts (Fig. 4). For example, in Mozambique, 88.5% of women with no ANC visits are in rural areas compared to 65.3% for women with at least four ANC visits. In South Africa, the proportion for women with no ANC visits residing in rural areas (24.2%) is lesser than the corresponding proportion for women with at least four ANC visits (38.0%).

**Fig. 5:**
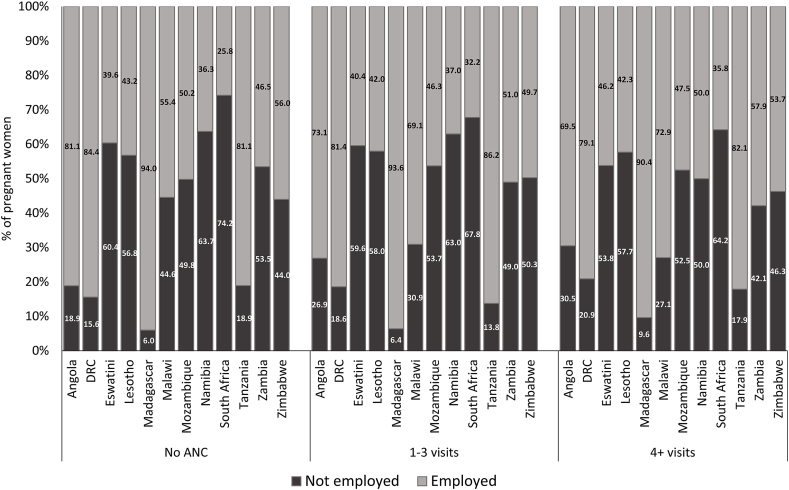
Distribution of ANC utilisation by in the Southern Africa Development Community countries employment status. *Note*: DRC = Democratic Republic of Congo.

Unlike the other SES measures (wealth index and education), the gradient for employment status is mixed (Fig. 5). In Angola, the Democratic Republic of Congo and Madagascar, the proportion of women without ANC visits who are not employed is higher than the corresponding proportion for women with at least four ANC visits. The reverse is seen in Eswatini, Malawi, Namibia and South Africa (Fig. 5). In Tanzania, the proportion of women with no ANC visits (18.9%) who are not employed is comparable to that for women with at least four ANC visits (17.9%), which is similar in Lesotho (56.8% and 57.7%, respectively).

**Fig. 6:**
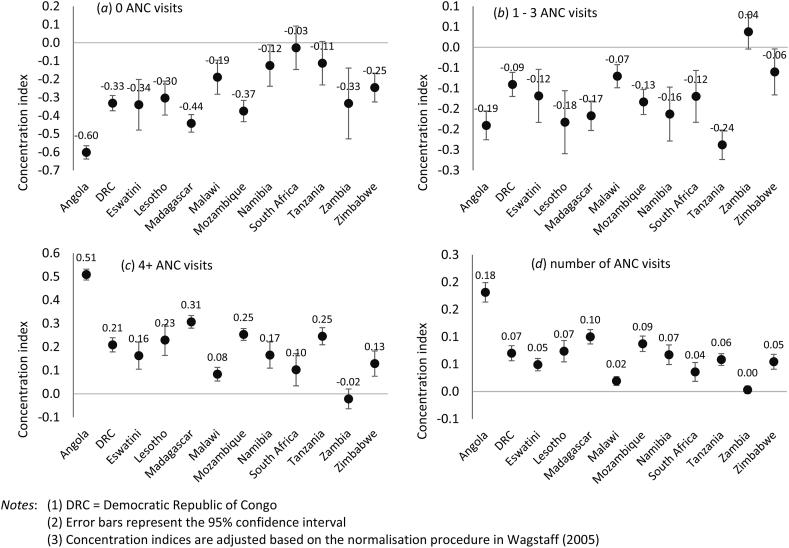
Socioeconomic inequality in antenatal care visits in the Southern Africa Development Community countries.

The Wagstaff normalised concentration indices in Fig. 6 (panel a) show that women from poorer households are significantly more likely to have no ANC visits than their wealthier counterparts. The concentration indices are negative and statistically significant at the 5% level, except South Africa and Tanzania, where the confidence intervals included zero. Overall, the negative concentration indices for panel (a) ranged between −0.03 (in South Africa with the least pro-poor distribution) and −0.6 (in Angola with the most pro-poor distribution). The results in Fig. 6 (panel b) also show that apart from Zambia, women from poorer backgrounds are significantly more likely to have between one and three ANC visits than wealthier women. In Zambia, the concentration index was positive, although this was not statistically significant as the 95% confidence interval included zero. The negative concentration indices in Fig. 6 (panel b) are statistically significant at the 5% level and range between −0.06 (in Zimbabwe, with the least pro-poor distribution) and −0.24 (in Tanzania, with the most pro-poor distribution). Interestingly, as shown in Fig. 6 (panel c), the concentration indices for the use of at least four ANC visits were all significantly pro-rich, except for Zambia, where it was negative and not statistically significant. This means that, in these countries, wealthier women are more likely to have attained at least four ANC visits than women from poorer backgrounds. The most pro-rich distribution of ANC 4+ utilisation was recorded for Angola (concentration index = 0.51), while Malawi had the least pro-rich distribution (concentration index = 0.08). Zambia's pro-poor distribution for ANC 4+ utilisation was expected because the concentration index was positive for between one and three ANC visits.

Socioeconomic inequalities in the number of ANC visits in Fig. 6 (panel d) show that, on average, wealthier women tend to have more ANC visits (ANC intensity) compared to women from poorer backgrounds as the concentration indices were positive and statistically significant for all countries, except Zambia where the index is approximately zero (<0.01). The concentration index estimated at close to zero for Zambia means no distinction exists in the number of ANC visits between women from poorer and wealthier backgrounds, at least in principle. For the countries with significantly positive concentration indices for the number of ANC visits, Angola's pro-rich concentration index is the highest (0.18), while Malawi's index is the lowest (0.02). These results show the existence of a socioeconomic gradient for the utilisation of antenatal services in the SADC countries, with women from poorer backgrounds recording fewer visits than their wealthier counterparts with significantly more visits.

**Fig. 7:**
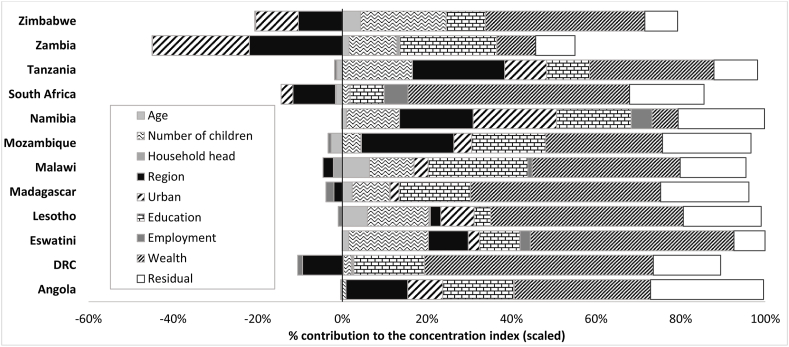
Factors explaining socioeconomic inequalities in ANC coverage, SADC countries.

After decomposing socioeconomic inequalities in ANC intensity (i.e. the number of ANC visits per woman), as shown in Fig. 7, major social determinants of health, including wealth, education, residency region, urban location, and a woman's number of children are major contributors explaining socioeconomic inequalities in ANC coverage in SADC countries. In most cases, these social determinants of ANC utilisation inequalities contribute significantly to the concentration of ANC utilisation among wealthier women (i.e., a positive concentration index). The residual component is also relatively prominent as this captures some factors not included in the model directly. Variables such as a woman's age and whether or not a woman is the head of the household did not explain socioeconomic disparities in ANC utilisation.

## 5 Discussion

ANC utilisation is critical for improving the health of mothers and their children (Shibre et al., 2020). However, ANC service coverage is still poor in many SADC countries. Using the previously recommended 4+ ANC visits for uncomplicated pregnancies, this paper shows a significant socioeconomic gradient in ANC utilisation in SADC countries as women from wealthier socioeconomic backgrounds record many more ANC visits, on average, than their poorer counterparts. Key social determinants of inequalities in ANC utilisation, including women's education, wealth and location, explain the significant socioeconomic inequalities in ANC service utilisation in SADC countries. Women with less than the previously recommended minimum of four ANC visits, including those with no ANC visits, tend to be predominantly from poorer socioeconomic backgrounds, as reflected in the significantly negative concentration indices reported in this paper.

This paper's finding is consistent with other studies where inadequate or complete lack of ANC service use is prevalent among poorer groups (Celik & Hotchkiss, 2000; Abor et al., 2011; Abekah-Nkrumah, 2019; Obse & Ataguba, 2021; Nwosu & Ataguba, 2019; Wabiri et al., 2013). While there are other significant determinants of maternal morbidity and mortality, the high proportion of pregnant women not receiving at least four ANC visits (Fig. 1), coupled with the significant socioeconomic gradient in ANC services utilisation in SADC countries, means that the region is not on track to reduce maternal and neonatal mortality substantially to meet the SDGs (SRHR Africa Trust, 2019). The neonatal mortality rate in many SADC countries still exceeds the SDG target of 12 deaths per 1,000 live births (SRHR Africa Trust, 2019). Apart from Mauritius and South Africa, where the issue is comparatively less pronounced, for instance, a challenge with improving ANC coverage in many SADC countries is the number and distribution of skilled health workers, especially in poorer regions, including rural locations (SRHR Africa Trust, 2019; Wabiri et al., 2013).

Teenage pregnancy rates are still high in some SADC countries. For example, Madagascar's teenage pregnancy rate exceeds 40% (SRHR Africa Trust, 2019). Fortunately, as found in this paper, age did not explain socioeconomic inequalities in ANC significantly. This may result from complex factors. Perhaps the Comprehensive Sexuality Education (CSE) adopted by SADC countries, based on the 2013 commitment of the Ministers of Education, Health and Youth from twenty East and Southern African countries, contributed to ameliorating the challenge (SRHR Africa Trust, 2019).

Promoting different interventions may have positively impacted maternal health inequalities in SADC countries, especially those with minimal socioeconomic inequalities in ANC coverage. For instance, partner involvement interventions implemented in Malawi in urban and rural areas (Kululanga et al., 2011) may have contributed to Malawi showing the least pro-rich inequality in 4+ ANC visits (concentration index = 0.08). Similarly, Zambia's “Safe Motherhood Action Groups” interventions, among others, which increased maternal services coverage among the poorest and most remote populations (Jacobs et al., 2018), may have contributed to the pro-poor inequalities in 4+ ANC visits, even though the pro-poor results were not statistically significant. A similar ‘Pillars of Safe Motherhood’ implemented in Zimbabwe focusing on ANC services, including the prevention of mother-to-child-transmission of HIV, nutrition, and ensuring these were made available to all pregnant women (Ministry of Health and Child Welfare, 2007) may be contributing to the relatively “small” pro-rich distribution of 4+ ANC visits in the country compared to the other SADC countries. South Africa, still facing some access barriers to utilising maternal health services (Silal et al., 2012), implemented free care for pregnant women and young children since 1996, contributing to significantly improving inequalities in ANC utilisation compared to other SADC countries. The results show that although about 6% of pregnant women in South Africa did not use any ANC service, this was not restricted to only women from poorer households (the concentration index for no ANC visits was −0.03, and this was not statistically different from zero). Similarly, the pro-rich inequality in ANC intensity in South Africa was very minimal (concentration index = 0.04).

Although free and subsidised maternal health services are prevalent in sub-Saharan Africa (Amo-Adjei and Anamaale Tuoyire, 2016; Ridde et al., 2014), Angola still records one of the highest proportions of women without ANC visits (>18%), with the majority (>73%) of these women residing in rural locations compared to 77% of women receiving 4+ ANC visits living in urban locations. The country also has the most pro-rich distribution of 4+ ANC utilisation (concentration index = 0.51) and ANC intensity (concentration index = 0.18) among the other SADC countries. Women without any ANC visits in Angola are predominantly from poorer families (concentration index = −0.60) and poorer localities (e.g., Cuanza sul), which may be due to many access barriers facing women from poorer socioeconomic backgrounds who cannot afford the medical and non-medical costs associated with using ANC (Shibre et al., 2020). This highlights the importance of implementing other complementary reforms to accompany free health services provision (Cleary et al., 2013), especially increasing the acceptability of services and covering transport costs.

Significant factors that explain socioeconomic inequalities in ANC service utilisation in the SADC region are essentially the social determinants of health (Commission on Social Determinants of Health, 2008; Ataguba et al., 2015; Umuhoza & Ataguba, 2018). These determinants include a woman's education, wealth, region of residence and urban/rural location. Obse and Ataguba (2021), for example, introduced the concepts of ANC deficits and surpluses and identified wealth, education and area of residency as critical determinants of socioeconomic inequalities in ANC utilisation in Africa. These results highlight the importance of investing in women's education and making ANC services available and affordable to pregnant women in rural and remote localities (McTavish et al., 2010). To reduce the socioeconomic inequalities in ANC utilisation found in this paper will require policies within each SADC country that address the key determinants driving inequalities. These policies should be context-specific and target vulnerable women from poorer socioeconomic backgrounds.

Beyond the social determinants of ANC inequalities identified in this paper, some supply-side factors may militate against the use of ANC services, including experience of poor service quality, staff shortage, poor staff attitude, long waiting time and long distance to the nearest health facility (Escamilla et al., 2018; Kaswa et al., 2018). These factors need urgent policy attention in countries where they represent a significant challenge to reduce maternal health inequalities. An integrated approach that can be strengthened in a country like Angola with very high ANC service utilisation inequality levels includes using a civil society organization like the Angolan Women Organization (OMA–Organização da Mulher Angolana), which is present in almost all neighbourhoods to promote women's access to general health care, education and also facilitate women's use of specific maternal health services such as antenatal, delivery and postnatal care (Rosário et al., 2019).

While reducing socioeconomic inequalities is essential, it is equally crucial to address the content and timing of ANC services to ensure that women receive adequate quality services (Hodgins & D'Agostino, 2014; Kyei et al., 2012; Beeckman et al., 2012). The timing of ANC visits, for instance, is crucial for reducing maternal and child deaths (Beeckman et al., 2012). Tanzania, where about 49% of pregnant women had less than four ANC visits, records only one in four women having their first ANC visit in the first trimester (SRHR Africa Trust, 2019). In South Africa, only 46% had their first ANC visit before 20 weeks (Wabiri et al., 2013). Tackling and improving the timing of ANC visits, addressing critical service access barriers and other significant social determinants of inequality in ANC utilisation and other supply-side factors will likely improve service utilisation among the poor who have prominently fewer visits than their wealthier counterparts. Some access barriers documented in the literature include high out-of-pocket costs, distance to a health facility and lack of spousal support (Ahinkorah et al., 2021; Fagbamigbe & Idemudia, 2015; Mutowo et al., 2021).

This study's strengths include using comparable national household surveys to assess socioeconomic inequalities in ANC service utilisation. Including ANC intensity in the analysis is another way to examine not only utilisation rates using a predetermined cut-off but the entire distribution of service utilisation. The study also presents inequalities in one of Africa's major regional blocks, the SADC region. However, using comparable national datasets, especially selecting the same variables for all countries, comes with a challenge. Using the same set of variables to decompose socioeconomic inequalities in ANC utilisation means we could not account for certain country-specific factors, which led to substantial unexplained components for many countries. Moreover, many of these variables are not in the datasets, which may well explain why the residual component in the decomposition analysis remained substantial. Although there are significant differences in the factors that affect ANC utilisation by countries (Simkhada et al., 2008), to ensure uniformity between countries, the decomposition analyses included variables that may be collinear in some country contexts. Country specific analyses are needed to include country-specific variables that may explain socioeconomic inequalities in ANC utilisation and reduce the residual component, recognising the possibility of collinearity among some variables. Some important country context variables for consideration in country-specific analyses to explain socioeconomic inequalities in ANC utilisation and reduce the unexplained component include cultural and religious beliefs and practices and exposure to media (Simkhada et al., 2008; Muhwava et al., 2016), with these issues being common among women from poorer backgrounds.

Another limitation is that the DHS datasets do not indicate complicated pregnancies, and all pregnancies were implicitly treated as uncomplicated in the analysis. The analysis in this paper also omitted variables like distance to the nearest health facility where ANC is delivered, which may determine whether a woman can use ANC service (Shibre et al., 2020) and the timing of all ANC visits. However, it is possible that including regional dummies and urban/rural localities will partly capture, for instance, the effects of distance to the facility. Also, this study did not include a variable to capture a woman's autonomy related to ANC service utilisation as this determines adequate access to ANC services. In Nigeria, for instance, religion, cultural beliefs and practices restrict some women from seeking health-related assistance during pregnancy (Rai et al., 2012). While this paper included a woman's employment status to capture the effects of a woman's autonomy, it was not a significant social determinant in the decomposition model. Also, because of the paucity of data, this paper did not account for the quality and content of ANC utilisation as every ANC utilisation was counted as the same.

For future research, it is critical to adopt a political economy approach that examines, among other things, colonial histories, local cultures and practices, political systems and how the current health delivery system evolved in countries and within the SADC region. Such an approach may be helpful to understand the persistent inequalities in ANC service utilisation within and between countries. Also, future analyses should pay attention to country-specific factors to better explain socioeconomic inequalities in ANC utilisation in countries and to understand how specific interventions and other contexts beyond interventions affect access to health services, leading to significant socioeconomic inequalities.

## 6 Conclusion

Access to antenatal care is vital for the health of women and their children. Unfortunately, women from poorer backgrounds are often left behind as they have fewer or no ANC visits compared to their wealthier counterparts. In the SADC region, the socioeconomic gradient in ANC service utilisation is prominent across all countries, irrespective of their income levels. Notably, the factors that explain the significant socioeconomic inequalities in ANC coverage reported in this paper point to the need to adopt an integrated strategy that involves other social service sectors working closely with the health sector. While it is crucial to address critical social determinants of inequalities in ANC service utilisation like women's education and economic well-being, there should be deliberate efforts within countries to reduce health service access barriers, including the availability, affordability and acceptability of health services (McIntyre et al., 2009; Simkhada et al., 2008). Addressing these multi-pronged and interrelated issues can potentially redress inequalities in ANC service coverage in the SADC region and reduce maternal morbidity and mortality. Countries should also ensure that a significant proportion of women attain at least 6 ANC *contacts* as recently recommended by the WHO, emphasising the quality, timing and content of service utilisation. Doing this will put the SADC region on the road to reaching key SDG targets and leave no woman behind.

## Ethical statement

This research uses anonymised publicly available data from the Demographic and Health Surveys. Therefore, there are no ethical issues. However, the study received ethics approval from the Human Research Ethics Committee at the University of Cape Town.

## Availability of data

The Demographic and Health Survey datasets used in this article are available in the DHS repository https://dhsprogram.com/data/available-datasets.cfm, and are accessible after registration on the website.

## Financial disclosure statement

Keolebogile Selebano acknowledges the funding received from the 10.13039/501100007112University of Cape Town through the 10.13039/501100001321NRF Masters Free Standing Scholarship. John E Ataguba acknowledges the funding received from the 10.13039/501100007112University of Cape Town through the post-SARChI award.

## Author statement

**Keolebogile Selebano** and **John E Ataguba** (contributed equally to this paper): Study conceptualisation, data acquisition and analysis, literature review, writing, editing and revising.

## Declaration of competing interest

There are no conflicts of interest to declare.

## References

[bib1] Abekah-Nkrumah G. (2019). Trends in utilisation and inequality in the use of reproductive health services in Sub-Saharan Africa. BMC Public Health.

[bib2] Abor P.A., Abekah‐Nkrumah G., Sakyi K., Adjasi C.K., Abor J. (2011). The socio‐economic determinants of maternal health care utilization in Ghana. International Journal of Social Economics.

[bib3] Adam T., Lim S.S., Mehta S., Bhutta Z.A., Fogstad H., Mathai M., Zupan J., Darmstadt G.L. (2005). Cost effectiveness analysis of strategies for maternal and neonatal health in developing countries. BMJ.

[bib4] Ahinkorah B.O., Ameyaw E.K., Seidu A.-A., Odusina E.K., Keetile M., Yaya S. (2021). Examining barriers to healthcare access and utilization of antenatal care services: Evidence from demographic health surveys in sub-Saharan Africa. BMC Health Services Research.

[bib5] Amo‐Adjei J., Anamaale Tuoyire D. (2016). Effects of planned, mistimed and unwanted pregnancies on the use of prenatal health services in sub‐Saharan Africa: A multicountry analysis of demographic and health survey data. Tropical Medicine and International Health.

[bib6] Andrade M.V., Noronha K., Singh A., Rodrigues C.G., Padmadas S.S. (2012). Antenatal care use in Brazil and India: Scale, outreach and socioeconomic inequality. Health & Place.

[bib7] Ataguba J.E. (2018). A reassessment of global antenatal care coverage for improving maternal health using sub-Saharan Africa as a case study. PLoS One.

[bib8] Ataguba J.E., Day C., McIntyre D. (2015). Explaining the role of the social determinants of health on health inequality in South Africa. Global Health Action.

[bib9] Beeckman K., Louckx F., Downe S., Putman K. (2012). The relationship between antenatal care and preterm birth: The importance of content of care. The European Journal of Public Health.

[bib10] Bilger M., Kruger E.J., Finkelstein E.A. (2017). Measuring socioeconomic inequality in obesity: Looking beyond the obesity threshold. Health Economics.

[bib11] Celik Y., Hotchkiss D.R. (2000). The socio-economic determinants of maternal health care utilization in Turkey. Social Science & Medicine.

[bib12] Cleary S., Birch S., Chimbindi N., Silal S., McIntyre D. (2013). Investigating the affordability of key health services in South Africa. Social Science & Medicine.

[bib13] Commission on Social Determinants of Health (2008). Final report of the commission on social determinants of health.

[bib14] Darmstadt G.L., Bhutta Z.A., Cousens S., Adam T., Walker N., De Bernis L., Team L.N.S.S. (2005). Evidence-based, cost-effective interventions: How many newborn babies can we save?. The Lancet.

[bib15] DHS Program (2021). https://dhsprogram.com/.

[bib16] Efron B. (1987). Better bootstrap confidence intervals. Journal of the American Statistical Association.

[bib17] Efron B., Tibshirani R. (1986). Bootstrap methods for standard errors, confidence intervals, and other measures of statistical accuracy. Statistical Science.

[bib18] Escamilla V., Calhoun L., Winston J., Speizer I.S. (2018). The role of distance and quality on facility selection for maternal and child health services in urban Kenya. Journal of Urban Health.

[bib19] Fagbamigbe A.F., Idemudia E.S. (2015). Barriers to antenatal care use in Nigeria: Evidences from non-users and implications for maternal health programming. BMC Pregnancy and Childbirth.

[bib20] Gil-González D., Carrasco-Portiño M., Ruiz M.T. (2006). Knowledge gaps in scientific literature on maternal mortality: A systematic review. Bulletin of the World Health Organization.

[bib21] Hodgins S., D'Agostino A. (2014). The quality–coverage gap in antenatal care: Toward better measurement of effective coverage. Globalization and Health: Science and Practice.

[bib22] Jacobs C., Michelo C., Moshabela M. (2018). Implementation of a community-based intervention in the most rural and remote districts of Zambia: A process evaluation of safe motherhood action groups. Implementation Science.

[bib23] Kakwani N., Wagstaff A., van Doorslaer E. (1997). Socioeconomic inequalities in health: Measurement, computation, and statistical inference. Journal of Econometrics.

[bib24] Kanyangarara M., Munos M.K., Walker N. (2017). Quality of antenatal care service provision in health facilities across sub–Saharan Africa: Evidence from nationally representative health facility assessments. Journal of Global Health.

[bib25] Kaswa R., Rupesinghe G.F., Longo-Mbenza B. (2018). Exploring the pregnant women's perspective of late booking of antenatal care services at Mbekweni Health Centre in Eastern Cape, South Africa. African Journal of Primary Health Care and Family Medicine.

[bib26] Kerber K.J., de Graft-Johnson J.E., Bhutta Z.A., Okong P., Starrs A., Lawn J.E. (2007). Continuum of care for maternal, newborn, and child health: From slogan to service delivery. The Lancet.

[bib27] Koroma M.M., Kamara S.S., Bangura E.A., Kamara M.A., Lokossou V., Keita N. (2017). The quality of free antenatal and delivery services in Northern Sierra Leone. Health Research Policy and Systems.

[bib28] Kululanga L.I., Sundby J., Chirwa E. (2011). Striving to promote male involvement in maternal health care in rural and urban settings in Malawi-a qualitative study. Reproductive Health.

[bib29] Kyei N.N.A., Chansa C., Gabrysch S. (2012). Quality of antenatal care in Zambia: A national assessment. BMC Pregnancy and Childbirth.

[bib30] Manthalu G., Yi D., Farrar S., Nkhoma D. (2016). The effect of user fee exemption on the utilization of maternal health care at mission health facilities in Malawi. Health Policy and Planning.

[bib31] Masiye F., Kaonga O., Kirigia J.M. (2016). Does user fee removal policy provide financial protection from catastrophic health care payments? Evidence from Zambia. PLoS One.

[bib32] McIntyre D., Thiede M., Birch S. (2009). Access as a policy-relevant concept in low- and middle-income countries. Health Policy, Economics and Law.

[bib33] McTavish S., Moore S., Harper S., Lynch J. (2010). National female literacy, individual socio-economic status, and maternal health care use in sub-Saharan Africa. Social Science & Medicine.

[bib34] Ministry of Health and Child Welfare (2007).

[bib35] Muhwava L.S., Morojele N., London L. (2016). Psychosocial factors associated with early initiation and frequency of antenatal care (ANC) visits in a rural and urban setting in South Africa: A cross-sectional survey. BMC Pregnancy and Childbirth.

[bib36] Mutowo J., Yazbek M., van der Wath A., Maree C. (2021). Barriers to using antenatal care services in a rural district in Zimbabwe. International Journal of Africa Nursing Sciences.

[bib37] Nagdeva D.A. (2009). Urban-rural differentials in maternal and child healthcare. Health and Population - Perspectives and Issues.

[bib38] Najafizada S.A.M., Bourgeault I.L., Labonté R. (2017). Social determinants of maternal health in Afghanistan: A review. Central Asian Journal of Global Health.

[bib39] Nwosu C.O., Ataguba J.E. (2019). Socioeconomic inequalities in maternal health service utilisation: A case of antenatal care in Nigeria using a decomposition approach. BMC Public Health.

[bib40] Obse A.G., Ataguba J.E. (2021). Explaining socioeconomic disparities and gaps in the use of antenatal care services in 36 countries in sub-Saharan Africa. Health Policy and Planning.

[bib41] Rai R.K., Singh P.K., Singh L. (2012). Utilization of maternal health care services among married adolescent women: Insights from the Nigeria demographic and health survey, 2008. Women's Health Issues.

[bib42] Ridde V., Morestin F. (2011). A scoping review of the literature on the abolition of user fees in health care services in Africa. Health Policy and Planning.

[bib43] Ridde V., Queuille L., Ndour M. (2014). Nine misconceptions about free healthcare in sub-Saharan Africa. Development Studies Research. An Open Access Journal.

[bib44] Rosário E.V.N., Gomes M.C., Brito M., Costa D. (2019). Determinants of maternal health care and birth outcome in the Dande Health and Demographic Surveillance System area, Angola. PLoS One.

[bib45] Rutstein S.O., Johnson K. (2004).

[bib46] Rutstein S.O., Rojas G. (2006).

[bib47] SADC (2021). https://www.sadc.int/member-states/.

[bib48] Sharma S.K., Sawangdee Y., Sirirassamee B. (2007). Access to health: women's status and utilization of maternal health services in Nepal. Journal of Biosocial Science.

[bib49] Shibre G., Zegeye B., Idriss-Wheeler D., Ahinkorah B.O., Oladimeji O., Yaya S. (2020). Socioeconomic and geographic variations in antenatal care coverage in Angola: Further analysis of the 2015 demographic and health survey. BMC Public Health.

[bib50] Silal S.P., Penn-Kekana L., Harris B., Birch S., McIntyre D. (2012). Exploring inequalities in access to and use of maternal health services in South Africa. BMC Health Services Research.

[bib51] Simkhada B., Teijlingen E.R.v., Porter M., Simkhada P. (2008). Factors affecting the utilization of antenatal care in developing countries: Systematic review of the literature. Journal of Advanced Nursing.

[bib52] SRHR Africa Trust (2019).

[bib53] StataCorp (2017).

[bib54] Umuhoza S.M., Ataguba J.E. (2018). Inequalities in health and health risk factors in the Southern African development community: Evidence from world health surveys. International Journal for Equity in Health.

[bib55] UNFPA (2012).

[bib56] United Nations (2015).

[bib57] United Nations Development Programme (2015).

[bib58] United Nations Development Programme (2015).

[bib59] Wabiri N., Chersich M., Zuma K., Blaauw D., Goudge J., Dwane N. (2013). Equity in maternal health in South Africa: Analysis of health service access and health status in a national household survey. PLoS One.

[bib60] Wagstaff A. (2005). The bounds of the concentration index when the variable of interest is binary, with an application to immunization inequality. Health Economics.

[bib61] Wagstaff A., Paci P., van Doorslaer E. (1991). On the measurement of inequalities in health. Social Science & Medicine.

[bib62] Wagstaff A., van Doorslaer E., Watanabe N. (2003). On decomposing the causes of health sector inequalities with an application to malnutrition inequalities in Vietnam. Journal of Econometrics.

[bib63] World Bank (2021). World data bank [online]. https://data.worldbank.org/indicator/.

[bib64] World Health Organization (2016).

[bib65] World Health Organization (2019).

[bib66] World Health Organization (2021). https://apps.who.int/nha/database/ViewData/Indicators/en.

[bib67] World Health Organization (2021). https://www.who.int/data/gho/data/themes/maternal-and-reproductive-health/maternal-mortality-country-profiles.

[bib68] Yaya S., Bishwajit G., Shah V. (2016). Wealth, education and urban–rural inequality and maternal healthcare service usage in Malawi. BMJ Global Health.

