# Supplementary material for: Decomposing socio-economic inequalities in antenatal care utilisation in 12 Southern African Development Community countries
Source: SSM Popul Health. 2021 Dec 16;17:101004. doi: 10.1016/j.ssmph.2021.101004 (PMC8703074; doi:10.1016/j.ssmph.2021.101004)
Supplement: Multimedia component 1 [file mmc1.docx]

**APPENDIX**

Full decomposition results

**Table A.1: Angola**

------------------------------------------------------------------------------------------

Conc. Index Elasticity Contribution

------------------------------------------------------------------------------------------

Age -0.000(0.003) 0.127**(0.061) -0.000(0.000)

No of children born -0.065**(0.007) -0.025(0.024) 0.002(0.002)

Household head -0.151**(0.017) 0.005(0.005) -0.001(0.001)

__Iregion_2 -0.009(0.047) 0.003**(0.001) -0.000(0.000)

__Iregion_3 -0.335**(0.044) -0.014*(0.008) 0.005*(0.003)

__Iregion_4 0.537**(0.018) 0.035**(0.011) 0.019**(0.006)

__Iregion_5 -0.107**(0.053) -0.001(0.001) 0.000(0.000)

__Iregion_6 -0.376**(0.037) -0.026*(0.013) 0.010*(0.005)

__Iregion_7 -0.099**(0.050) 0.001(0.003) -0.000(0.000)

__Iregion_8 -0.208**(0.053) -0.008**(0.004) 0.002*(0.001)

__Iregion_9 -0.113**(0.043) 0.004(0.006) -0.000 (0.001)

__Iregion_10 -0.225**(0.047) 0.016**(0.004) -0.004**(0.001)

__Iregion_11 -0.408**(0.030) 0.002(0.004) -0.001(0.001)

__Iregion_12 -0.305**(0.061) -0.007**(0.003) 0.002*(0.001)

__Iregion_13 -0.225**(0.039) -0.004*(0.002) 0.001(0.001)

__Iregion_14 -0.040(0.062) 0.001(0.001) -0.000(0.000)

__Iregion_15 -0.410**(0.051) 0.001(0.007) -0.000(0.003)

__Iregion_16 -0.545**(0.049) 0.010**(0.002) -0.006**(0.001)

__Iregion_17 -0.074(0.055) 0.002(0.001) -0.000(0.000)

__Iregion_18 -0.056(0.054) 0.001(0.001) -0.000(0.000)

__Iurban_2 0.322**(0.010) 0.047**(0.020) 0.015**(0.006)

__Ieducation_2 -0.117**(0.018) 0.072**(0.008) -0.008**(0.002)

__Ieducation_3 0.397**(0.012) 0.077**(0.006) 0.031**(0.002)

__Ieducation_4 0.865**(0.017) 0.010**(0.002) 0.008**(0.001)

__Iemployment_2 -0.066**(0.009) -0.005(0.019) 0.000(0.001)

__Iquintile_2 -0.385**(0.020) 0.046**(0.009) -0.018**(0.004)

__Iquintile_3 0.049**(0.023) 0.059**(0.008) 0.003**(0.001)

__Iquintile_4 0.464**(0.022) 0.061**(0.008) 0.028**(0.004)

__Iquintile_5 0.833**(0.011) 0.054**(0.006) 0.045**(0.005)

Residual 0.049**(0.007)

**Total 0.181**(0.007)**

Region 0.027**(0.009)

Urban residence 0.015**(0.006)

Highest education level 0.031**(0.003)

Employment status 0.000(0.001)

Wealth quintiles 0.059**(0.006)

------------------------------------------------------------------------------------------

Observations 8,839

------------------------------------------------------------------------------------------

Standard errors in parentheses

* p<0.1, ** p<0.05

*Note*: __Iregion_1 = Cabinda; __Iregion_2 = Zaire; __Iregion_3 = Uíge; __Iregion_4 = Luanda; __Iregion_5 = Cuanza Norte; __Iregion_6 = Cuanza Sul; __Iregion_7 = Malanje; __Iregion_8 = Lunda Norte; __Iregion_9 = Benguela; __Iregion_10 = Huambo; __Iregion_11 = Bié; __Iregion_12 = Moxico; __Iregion_13 = Cuando Cubango; __Iregion_14 = Namibe; __Iregion_15 = Huíla; __Iregion_16 = Cunene; __Iregion_17 = Lunda Sul; __Iregion_18 = Bengo

__Ieducation_1 = no formal education; __Ieducation_2 = Primary education; __Ieducation_3 = Secondary education; __Ieducation_4 = Tertiary education

__Iurban_1 = urban location; __Iurban_2 = rural location

__Iemployment_1 = Not employed; __Iemployment_2 = employed

__Iquintile_1 = Q1(poorest quintile); __Iquintile_2 = Q2; __Iquintile_3 = Q3; __Iquintile_4 = Q4; __Iquintile_5 = Q5(richest quintile)

**Table A.2: Democratic Republic of Congo**

------------------------------------------------------------------------------------------

Conc. Index Elasticity Contribution

------------------------------------------------------------------------------------------

Age 0.002(0.002) 0.150**(0.048) 0.000(0.000)

No. of children born -0.016**(0.006) -0.098**(0.021) 0.002**(0.001)

Household head -0.177**(0.020) -0.002(0.004) 0.000(0.001)

__Iregion_2 -0.202**(0.042) 0.004(0.011) -0.001(0.002)

__Iregion_3 0.250**(0.094) -0.007*(0.004) -0.002(0.001)

__Iregion_4 -0.336**(0.042) 0.006(0.008) -0.002(0.003)

__Iregion_5 -0.213**(0.085) 0.001(0.004) -0.000(0.001)

__Iregion_6 -0.015(0.067) -0.008(0.006) 0.000(0.001)

__Iregion_7 0.149*(0.088) -0.012**(0.006) -0.002(0.002)

__Iregion_8 -0.075(0.064) -0.003(0.003) 0.000(0.000)

__Iregion_9 0.052(0.098) 0.007(0.005) 0.000(0.001)

__Iregion_10 -0.138**(0.061) 0.005(0.005) -0.001(0.001)

__Iregion_11 0.156**(0.066) -0.011*(0.007) -0.002(0.001)

__Iurban_2 0.542**(0.027) 0.000(0.008) 0.000(0.004)

__Ieducation_2 -0.149**(0.015) 0.013(0.013) -0.002(0.002)

__Ieducation_3 0.276**(0.016) 0.048**(0.011) 0.013**(0.003)

__Ieducation_4 0.805**(0.032) 0.004**(0.001) 0.003**(0.001)

__Iemployment_2 -0.042**(0.007) 0.024(0.015) -0.001(0.001)

__Iquintile_2 -0.349**(0.031) 0.026**(0.006) -0.009**(0.002)

__Iquintile_3 0.072*(0.043) 0.034**(0.008) 0.002(0.002)

__Iquintile_4 0.462**(0.042) 0.038**(0.007) 0.018**(0.004)

__Iquintile_5 0.823**(0.021) 0.045**(0.008) 0.037**(0.006)

Residual 0.014**(0.003)

**Total 0.070**(0.007)**

Region -0.008*(0.005)

Urban residence 0.000(0.004)

Highest education level 0.015**(0.002)

Employment status -0.001(0.001)

Wealth quintiles 0.048**(0.007)

------------------------------------------------------------------------------------------

Observations 11,214

------------------------------------------------------------------------------------------

Standard errors in parentheses

* p<0.1, ** p<0.05

*Note*: __Iregion_1 = Kinshasa; __Iregion_2 = Bandundu; __Iregion_3 = Bas-Congo; __Iregion_4 = Équateur; __Iregion_5 = Kasaï-Occidental; __Iregion_6 = Kasaï-oriental; __Iregion_7 = Katanga; __Iregion_8 = Maniema; __Iregion_9 = Nord-Kivu; __Iregion_10 = Orientale; __Iregion_11 = Sud-Kivu

__Ieducation_1 = no formal education; __Ieducation_2 = Primary education; __Ieducation_3 = Secondary education; __Ieducation_4 = Tertiary education

__Iurban_1 = urban location; __Iurban_2 = rural location

__Iemployment_1 = Not employed; __Iemployment_2 = employed

__Iquintile_1 = Q1(poorest quintile); __Iquintile_2 = Q2; __Iquintile_3 = Q3; __Iquintile_4 = Q4; __Iquintile_5 = Q5(richest quintile)

**Table A.3: Eswatini**

------------------------------------------------------------------------------------------

Conc. Index Elasticity Contribution

------------------------------------------------------------------------------------------

Age 0.002(0.003) 0.357**(0.069) 0.001(0.001)

No. of children born -0.082**(0.009) -0.113**(0.025) 0.009**(0.002)

Household head -0.003(0.024) 0.009(0.006) -0.000(0.000)

__Iregion_2 0.161**(0.046) 0.011(0.008) 0.002(0.001)

__Iregion_3 -0.165**(0.040) -0.010(0.007) 0.002(0.001)

__Iregion_4 -0.172**(0.054) -0.008(0.007) 0.001(0.001)

__Iurban_2 0.513**(0.033) 0.003(0.006) 0.001(0.003)

__Ieducation_2 -0.240**(0.020) -0.003(0.013) 0.001(0.003)

__Ieducation_3 0.118**(0.018) -0.003(0.019) -0.000(0.002)

__Ieducation_4 0.767**(0.023) 0.006**(0.003) 0.004**(0.002)

__Iemployment_2 0.147**(0.017) 0.008(0.009) 0.001(0.001)

__Iquintile_2 -0.430**(0.036) -0.001(0.007) 0.000(0.003)

__Iquintile_3 -0.031(0.043) 0.013**(0.006) -0.000(0.001)

__Iquintile_4 0.372**(0.039) 0.013**(0.006) 0.005**(0.002)

__Iquintile_5 0.789**(0.018) 0.024**(0.007) 0.019**(0.006)

Residual 0.004**(0.002)

**Total 0.049**(0.005)**

Region 0.005**(0.002)

Urban residence 0.001(0.003)

Highest education level 0.005*(0.003)

Employment status 0.001(0.001)

Wealth quintiles 0.024**(0.006)

------------------------------------------------------------------------------------------

Observations 2,069

------------------------------------------------------------------------------------------

Standard errors in parentheses

* p<0.1, ** p<0.05

*Note*: __Iregion1 = Hhohho; __Iregion2 = Manzini; __Iregion3 = Shiselweni; __Iregion4 = Lubombo

__Ieducation_1 = no formal education; __Ieducation_2 = Primary education; __Ieducation_3 = Secondary education; __Ieducation_4 = Tertiary education

__Iurban_1 = urban location; __Iurban_2 = rural location

__Iemployment_1 = Not employed; __Iemployment_2 = employed

__Iquintile_1 = Q1(poorest quintile); __Iquintile_2 = Q2; __Iquintile_3 = Q3; __Iquintile_4 = Q4; __Iquintile_5 = Q5(richest quintile)

**Table A.4: Lesotho**

------------------------------------------------------------------------------------------

Conc. Index Elasticity Contribution

------------------------------------------------------------------------------------------

Age 0.010**(0.003) 0.428**(0.071) 0.004**(0.002)

No. of children born -0.076**(0.009) -0.143**(0.029) 0.011**(0.002)

Household head -0.024(0.044) -0.011**(0.004) 0.000(0.001)

__Iregion_2 0.094*(0.050) -0.003(0.008) -0.000(0.001)

__Iregion_3 0.150**(0.065) 0.000(0.007) 0.000(0.001)

__Iregion_4 0.284**(0.039) 0.017(0.012) 0.005(0.003)

__Iregion_5 0.206**(0.048) 0.006(0.004) 0.001(0.001)

__Iregion_6 -0.212**(0.075) 0.000(0.004) -0.000(0.001)

__Iregion_7 -0.108(0.068) -0.003(0.003) 0.000(0.000)

__Iregion_8 -0.283**(0.065) 0.003**(0.001) -0.001**(0.000)

__Iregion_9 -0.510**(0.042) 0.004(0.003) -0.002(0.002)

__Iregion_10 -0.518**(0.053) 0.002(0.005) -0.001(0.003)

__Iurban_2 0.547**(0.021) 0.011(0.009) 0.006(0.005)

__Ieducation_2 -0.282**(0.018) 0.041(0.041) -0.012(0.012)

__Ieducation_3 0.160**(0.015) 0.040(0.042) 0.006(0.007)

__Ieducation_4 0.720**(0.024) 0.011*(0.006) 0.008*(0.004)

__Iemployment_2 0.203**(0.015) -0.004(0.011) -0.001(0.002)

__Iquintile_2 -0.406**(0.032) 0.003(0.008) -0.001(0.003)

__Iquintile_3 -0.004(0.036) 0.009(0.007) -0.000(0.000)

__Iquintile_4 0.407**(0.032) 0.014(0.008) 0.006(0.004)

__Iquintile_5 0.807**(0.015) 0.037**(0.009) 0.030**(0.007)

residual 0.014**(0.004)

**Total 0.073**(0.010)**

Region 0.002(0.004)

Urban residence 0.006(0.005)

Highest education level 0.003(0.004)

Employment status -0.001(0.002)

Wealth quintiles 0.034**(0.008)

------------------------------------------------------------------------------------------

Observations 2,515

------------------------------------------------------------------------------------------

Standard errors in parentheses

* p<0.1, ** p<0.05

Note: __Iregion1 = Botha-Bothe; __Iregion2 = Leribe; __Iregion3 = Berea; __Iregion4 = Maseru; __Iregion5 = Mafeteng; __Iregion6 = Mohale's Hoek; __Iregion7 = Quthing; __Iregion8 = Qacha's-nek; __Iregion9 = Mokhotlong; __Iregion10 = Thaba Tseka

__Ieducation_1 = no formal education; __Ieducation_2 = Primary education; __Ieducation_3 = Secondary education; __Ieducation_4 = Tertiary education

__Iurban_1 = urban location; __Iurban_2 = rural location

__Iemployment_1 = Not employed; __Iemployment_2 = employed

__Iquintile_1 = Q1(poorest quintile); __Iquintile_2 = Q2; __Iquintile_3 = Q3; __Iquintile_4 = Q4; __Iquintile_5 = Q5(richest quintile)

**Table A.5: Madagascar**

------------------------------------------------------------------------------------------

Conc. Index Elasticity Contribution

------------------------------------------------------------------------------------------

Age 0.009**(0.002) 0.294**(0.062) 0.003** (0.001)

No. of children born -0.078**(0.006) -0.123**(0.022) 0.010**(0.002)

Household head -0.039(0.025) -0.004*(0.002) 0.000(0.000)

__Iregion_2 0.117**(0.058) -0.016**(0.005) -0.002*(0.001)

__Iregion_3 0.185**(0.054) -0.001(0.001) -0.000(0.000)

__Iregion_4 0.136**(0.051) -0.005**(0.002) -0.001**(0.000)

__Iregion_5 0.004(0.060) -0.008**(0.003) -0.000(0.001)

__Iregion_6 -0.022(0.081) -0.006**(0.002) 0.000(0.000)

__Iregion_7 -0.423**(0.033) 0.004(0.003) -0.002(0.001)

__Iregion_8 -0.266**(0.070) 0.000(0.001) -0.000(0.000)

__Iregion_9 -0.449**(0.043) -0.001(0.004) 0.000(0.002)

__Iregion_10 0.166*(0.088) -0.003(0.003) -0.000(0.000)

__Iregion_11 -0.104**(0.043) 0.000(0.002) -0.000(0.000)

__Iregion_12 0.240**(0.044) -0.007**(0.002) -0.002**(0.001)

__Iregion_13 0.104(0.092) 0.001(0.001) 0.000(0.000)

__Iregion_14 -0.262**(0.053) 0.004(0.003) -0.001(0.001)

__Iregion_15 -0.362**(0.080) -0.001(0.001) 0.000(0.000)

__Iregion_16 -0.472**(0.076) -0.002(0.001) 0.001(0.001)

__Iregion_17 -0.362**(0.079) -0.003(0.004) 0.001(0.001)

__Iregion_18 -0.462**(0.046) -0.002(0.002) 0.001(0.001)

__Iregion_19 -0.332**(0.073) -0.003(0.002) 0.001(0.001)

__Iregion_20 -0.250**(0.086) 0.000(0.001) -0.000(0.000)

__Iregion_21 0.104(0.085) 0.000(0.001) 0.000(0.000)

__Iregion_22 0.063(0.063) 0.001(0.002) 0.000(0.000)

__Iurban_2 0.715**(0.016) 0.003(0.003) 0.002(0.002)

__Ieducation_2 -0.042**(0.010) 0.063**(0.011) -0.003**(0.001)

__Ieducation_3 0.506**(0.012) 0.034**(0.004) 0.017**(0.002)

__Ieducation_4 0.925**(0.012) 0.004**(0.001) 0.004**(0.001)

__Iemployment_2 -0.037**(0.004) 0.056**(0.023) -0.002**(0.001)

__Iquintile_2 -0.316**(0.021) 0.024**(0.005) -0.008**(0.002)

__Iquintile_3 0.100**(0.025) 0.031**(0.005) 0.003**(0.001)

__Iquintile_4 0.486**(0.023) 0.034**(0.005) 0.017**(0.002)

__Iquintile_5 0.836**(0.011) 0.044**(0.005) 0.037**(0.004)

Residual 0.023**(0.002)

**Total 0.100**(0.006)**

Region -0.002(0.004)

Urban residence 0.002(0.002)

Highest education level 0.018**(0.002)

Employment status -0.002**(0.001)

Wealth quintiles 0.049**(0.005)

------------------------------------------------------------------------------------------

Observations 8,469

------------------------------------------------------------------------------------------

Standard errors in parentheses

* p<0.1, ** p<0.05

Note: __Iregion1 = Analamanga; __Iregion2 = Vakinankaratra; __Iregion3 = Itasy; __Iregion4 = Bongolava; __Iregion5 = Haute Matsiatra; __Iregion6 = Anamoroni'i Mania; __Iregion7 = Vatovavy Fitovinany; __Iregion8 = Ihorombe; __Iregion9 = Atsimo Atsinanana; __Iregion10 = Atsinanana; __Iregion11 = Analanjirofo; __Iregion12 = Alaotra Mangoro; __Iregion13 = Boeny; __Iregion14 = Sofia; __Iregion15 = Betsiboka; __Iregion16 = Melaky; __Iregion17 = Atsimo Andrefana; __Iregion18 = Androy; __Iregion19 = Anosy; __Iregion20 = Menabe; __Iregion21 = Diana; __Iregion22 = Sava

__Ieducation_1 = no formal education; __Ieducation_2 = Primary education; __Ieducation_3 = Secondary education; __Ieducation_4 = Tertiary education

__Iurban_1 = urban location; __Iurban_2 = rural location

__Iemployment_1 = Not employed; __Iemployment_2 = employed

__Iquintile_1 = Q1(poorest quintile); __Iquintile_2 = Q2; __Iquintile_3 = Q3; __Iquintile_4 = Q4; __Iquintile_5 = Q5(richest quintile)

**Table A.6: Malawi**

------------------------------------------------------------------------------------------

Conc. Index Elasticity Contribution

------------------------------------------------------------------------------------------

Age 0.006**(0.002) 0.225**(0.035) 0.001**(0.000)

No. of children born -0.036**(0.004) -0.062**(0.031) 0.002**(0.001)

Household head -0.256**(0.011) 0.002(0.002) -0.000(0.001)

__Iregion_2 -0.044**(0.012) 0.012*(0.006) -0.001*(0.000)

__Iregion_3 -0.005(0.011) -0.000(0.007) 0.000(0.000)

__Iurban_2 0.689**(0.017) 0.001(0.003) 0.001(0.002)

__Ieducation_2 -0.096**(0.007) 0.035**(0.010) -0.003**(0.001)

__Ieducation_3 0.407**(0.013) 0.011**(0.004) 0.005**(0.001)

__Ieducation_4 0.890**(0.020) 0.004**(0.021) 0.004**(0.001)

__Iemployment_2 -0.029**(0.005) -0.007(0.012) 0.000(0.000)

__Iquintile_2 -0.311**(0.013) 0.004(0.003) -0.001(0.001)

__Iquintile_3 0.099**(0.015) 0.001(0.003) 0.000(0.000)

__Iquintile_4 0.472**(0.012) 0.002(0.003) 0.001(0.001)

__Iquintile_5 0.826**(0.006) 0.009**(0.003) 0.008**(0.002)

Residual 0.003**(0.001)

**Total 0.019**(0.004)**

Region -0.001(0.000)

Urban 0.001(0.002)

Highest education level 0.005**(0.002)

Employment status 0.000(0.000)

Wealth quintiles 0.007**(0.003)

------------------------------------------------------------------------------------------

Observations 13,389

------------------------------------------------------------------------------------------

Standard errors in parentheses

* p<0.1, ** p<0.05

Note: __Iregion1 = Northern region; __Iregion2 = Central region; __Iregion3 = Southern region

__Ieducation_1 = no formal education; __Ieducation_2 = Primary education; __Ieducation_3 = Secondary education; __Ieducation_4 = Tertiary education

__Iurban_1 = urban location; __Iurban_2 = rural location

__Iemployment_1 = Not employed; __Iemployment_2 = employed

__Iquintile_1 = Q1(poorest quintile); __Iquintile_2 = Q2; __Iquintile_3 = Q3; __Iquintile_4 = Q4; __Iquintile_5 = Q5(richest quintile)

**Table A.7: Mozambique**

------------------------------------------------------------------------------------------

Conc. Index Elasticity Contribution

------------------------------------------------------------------------------------------

Age -0.017**(0.002) 0.142**(0.049) -0.002**(0.001)

No. of children born -0.071**(0.006) -0.058**(0.021) 0.004**(0.002)

Household head -0.038**(0.016) -0.003(0.004) 0.000(0.000)

__Iregion_2 -0.286**(0.039) 0.014**(0.003) -0.004**(0.001)

__Iregion_3 -0.188**(0.046) -0.001(0.007) 0.000(0.001)

__Iregion_4 -0.388**(0.039) -0.006(0.012) 0.002(0.005)

__Iregion_5 -0.121**(0.043) 0.007(0.007) -0.001(0.001)

__Iregion_6 0.227**(0.037) 0.021**(0.002) 0.005**(0.001)

__Iregion_7 0.082**(0.041) 0.013**(0.004) 0.001*(0.001)

__Iregion_8 0.299**(0.044) 0.007**(0.002) 0.002**(0.001)

__Iregion_9 0.412**(0.037) 0.010**(0.002) 0.004**(0.001)

__Iregion_10 0.705**(0.029) 0.008**(0.002) 0.006**(0.002)

__Iregion_11 0.861**(0.010) 0.006**(0.001) 0.005**(0.001)

__Iurban_2 0.476**(0.025) 0.008(0.006) 0.004(0.003)

__Ieducation_2 0.015(0.012) 0.042**(0.009) 0.001(0.000)

__Ieducation_3 0.646**(0.016) 0.021**(0.003) 0.013**(0.002)

__Ieducation_4 0.943**(0.008) 0.003**(0.001) 0.002**(0.000)

__Iemployment_2 -0.043**(0.010) 0.016**(0.008) -0.001*(0.000)

__Iquintile_2 -0.348**(0.024) 0.009(0.007) -0.003(0.002)

__Iquintile_3 0.055**(0.025) 0.015**(0.006) 0.001*(0.000)

__Iquintile_4 0.454**(0.019) 0.018**(0.006) 0.008**(0.003)

__Iquintile_5 0.828**(0.008) 0.024**(0.006) 0.020**(0.005)

Residual 0.020**(0.003)

**Total 0.088**(0.007)**

Region 0.021**(0.005)

Urban residence 0.004(0.003)

Highest education level 0.016**(0.002)

Employment status -0.001*(0.000)

Wealth quintiles 0.026**(0.006)

------------------------------------------------------------------------------------------

Observations 7,485

------------------------------------------------------------------------------------------

Standard errors in parentheses

* p<0.1, ** p<0.05

Note: __Iregion1 = Niassa; __Iregion2 = Cabo Delgado; __Iregion3 = Nampula; __Iregion4 = Zambezia; __Iregion5 = Tete; __Iregion6 = Manica; __Iregion7 = Sofala; __Iregion8 = Inhambane; __Iregion9 = Gaza; __Iregion10 = Maputo Provincia; __Iregion11 = Maputo Cidade

__Ieducation_1 = no formal education; __Ieducation_2 = Primary education; __Ieducation_3 = Secondary education; __Ieducation_4 = Tertiary education

__Iurban_1 = urban location; __Iurban_2 = rural location

__Iemployment_1 = Not employed; __Iemployment_2 = employed

__Iquintile_1 = Q1(poorest quintile); __Iquintile_2 = Q2; __Iquintile_3 = Q3; __Iquintile_4 = Q4; __Iquintile_5 = Q5(richest quintile)

**Table A.8: Namibia**

------------------------------------------------------------------------------------------

Conc. Index Elasticity Contribution

------------------------------------------------------------------------------------------

Age 0.002(0.003) 0.366**(0.084) 0.001(0.001)

No. of children born -0.113**(0.008) -0.075**(0.031) 0.008**(0.003)

Household head -0.010(0.023) 0.011(0.007) -0.000(0.000)

__Iregion_2 0.456**(0.060) 0.007*(0.004) 0.003*(0.002)

__Iregion_3 0.258**(0.071) 0.002(0.002) 0.000(0.000)

__Iregion_4 0.274**(0.064) 0.010**(0.002) 0.003**(0.001)

__Iregion_5 -0.410**(0.035) -0.001(0.008) 0.001(0.003)

__Iregion_6 0.464**(0.046) 0.043**(0.012) 0.020**(0.005)

__Iregion_7 -0.078(0.061) 0.011**(0.002) -0.001(0.001)

__Iregion_8 -0.426**(0.063) 0.023**(0.004) -0.010**(0.002)

__Iregion_9 -0.092*(0.053) 0.005**(0.002) -0.000(0.000)

__Iregion_10 -0.228**(0.048) 0.021**(0.004) -0.005**(0.001)

__Iregion_11 0.082(0.055) 0.023**(0.003) 0.002(0.001)

__Iregion_12 -0.189**(0.061) 0.018**(0.004) -0.003**(0.001)

__Iregion_13 0.186**(0.058) 0.010**(0.003) 0.002**(0.001)

__Iurban_2 0.350**(0.017) 0.038**(0.016) 0.013**(0.006)

__Ieducation_2 -0.377**(0.021) 0.043**(0.012) -0.016**(0.005)

__Ieducation_3 0.083**(0.011) 0.164**(0.027) 0.014**(0.003)

__Ieducation_4 0.696**(0.022) 0.021**(0.004) 0.014**(0.003)

__Iemployment_2 0.200**(0.015) 0.017(0.012) 0.003(0.002)

__Iquintile_2 -0.391**(0.024) -0.005(0.009) 0.002(0.004)

__Iquintile_3 0.007(0.032) -0.008(0.008) -0.000(0.000)

__Iquintile_4 0.429**(0.030) -0.005(0.011) -0.002(0.005)

__Iquintile_5 0.825**(0.015) 0.005(0.011) 0.004(0.009)

Residual 0.014**(0.003)

**Total 0.067**(0.009)**

Region 0.012(0.007)

Urban residence 0.013**(0.006)

Highest education level 0.012**(0.003)

Employment status 0.003(0.002)

Wealth quintiles 0.004(0.012)

------------------------------------------------------------------------------------------

Observations 3,116

------------------------------------------------------------------------------------------

Standard errors in parentheses

* p<0.1, ** p<0.05

*Note*: __Iregion1 = Caprivi; __Iregion2 = Erongo; __Iregion3 = Hardap; __Iregion4 = Karas; __Iregion5 = Kavango; __Iregion6 = Khomas; __Iregion7 = Kunene; __Iregion8 = Ohangwena; __Iregion9 = Omaheke; __Iregion10 = Omusati; __Iregion11 = Oshana; __Iregion12 = Oshikoto; __Iregion13 = Otjozondjupa

__Ieducation_1 = no formal education; __Ieducation_2 = Primary education; __Ieducation_3 = Secondary education; __Ieducation_4 = Tertiary education

__Iurban_1 = urban location; __Iurban_2 = rural location

__Iemployment_1 = Not employed; __Iemployment_2 = employed

__Iquintile_1 = Q1(poorest quintile); __Iquintile_2 = Q2; __Iquintile_3 = Q3; __Iquintile_4 = Q4; __Iquintile_5 = Q5(richest quintile)

**Table A.9: South Africa**

------------------------------------------------------------------------------------------

Conc. Index Elasticity Contribution

------------------------------------------------------------------------------------------

Age 0.013**(0.003) -0.058(0.064) -0.001(0.001)

No. of children -0.039**(0.007) -0.024(0.028) 0.001(0.001)

Household head -0.089**(0.028) 0.001(0.006) -0.000(0.001)

__Iregion_2 -0.270**(0.059) -0.016**(0.008) 0.004*(0.002)

__Iregion_3 0.056(0.002) -0.006**(0.002) -0.000(0.000)

__Iregion_4 0.213**(0.047) -0.002(0.003) -0.000(0.001)

__Iregion_5 -0.099*(0.059) -0.044**(0.013) 0.004(0.003)

__Iregion_6 -0.042(0.045) -0.008(0.005) 0.000(0.000)

__Iregion_7 0.171**(0.053) -0.130**(0.028) -0.022**(0.007)

__Iregion_8 -0.139*(0.072) -0.026**(0.007) 0.004*(0.002)

__Iregion_9 -0.292**(0.043) -0.017**(0.007) 0.005**(0.002)

__Iurban_2 0.213**(0.018) -0.007(0.018) -0.001(0.004)

__Ieducation_2 -0.431**(0.034) 0.002(0.007) -0.001(0.003)

__Ieducation_3 -0.027**(0.010) -0.024(0.064) 0.001(0.002)

__Ieducation_4 0.521**(0.032) 0.008(0.008) 0.004(0.004)

__Iemployment_2 0.184**(0.020) 0.016*(0.009) 0.003*(0.002)

__Iquintile_2 -0.324**(0.034) 0.006(0.009) -0.002(0.003)

__Iquintile_3 0.140**(0.036) 0.016*(0.008) 0.002(0.001)

__Iquintile_4 0.544**(0.029) 0.013(0.008) 0.007(0.004)

__Iquintile_5 0.864**(0.013) 0.023**(0.007) 0.020**(0.006)

Residual 0.009**(0.002)

**Total 0.037**(0.009)**

Region -0.005(0.005)

Urban residence -0.001(0.004)

Highest education level 0.004(0.003)

Employment status 0.003*(0.002)

Wealth quintiles 0.027**(0.008)

------------------------------------------------------------------------------------------

Observations 2,825

------------------------------------------------------------------------------------------

Standard errors in parentheses

* p<0.1, ** p<0.05

Note: __Iregion1 = Western cape; __Iregion2 = Eastern cape; __Iregion3 = Northern cape; __Iregion4 = Free state; __Iregion5 = Kwazulu-Natal; __Iregion6 = North west; __Iregion7 = Gauteng; __Iregion8 = Mpumalanga; __Iregion9 = Limpopo

__Ieducation_1 = no formal education; __Ieducation_2 = Primary education; __Ieducation_3 = Secondary education; __Ieducation_4 = Tertiary education

__Iurban_1 = urban location; __Iurban_2 = rural location

__Iemployment_1 = Not employed; __Iemployment_2 = employed

__Iquintile_1 = Q1(poorest quintile); __Iquintile_2 = Q2; __Iquintile_3 = Q3; __Iquintile_4 = Q4; __Iquintile_5 = Q5(richest quintile)

**Table A.10: Tanzania**

------------------------------------------------------------------------------------------

Conc. Index Elasticity Contribution

------------------------------------------------------------------------------------------

Age -0.004*(0.002) 0.203**(0.042) -0.001*(0.000)

No. of children born -0.107**(0.005) -0.094**(0.018) 0.010**(0.002)

Household head 0.046(0.033) -0.000(0.002) -0.000(0.000)

__Iregion_2 0.020(0.128) -0.003(0.002) -0.000(0.000)

__Iregion_3 0.387**(0.074) -0.001(0.001) -0.000(0.000)

__Iregion_4 0.148*(0.087) 0.002(0.002) 0.000(0.000)

__Iregion_5 0.075(0.081) 0.005**(0.002) 0.000(0.000)

__Iregion_6 0.121*(0.066) 0.002**(0.001) 0.000*(0.000)

__Iregion_7 0.765**(0.016) 0.008**(0.003) 0.006**(0.002)

__Iregion_8 -0.126**(0.059) 0.000(0.001) -0.000(0.000)

__Iregion_9 -0.124**(0.054) -0.001(0.001) 0.000(0.000)

__Iregion_10 0.039(0.080) -0.004**(0.002) -0.000(0.000)

__Iregion_11 0.255**(0.078) -0.001(0.001) -0.000(0.000)

__Iregion_12 0.022(0.074) -0.007*(0.004) -0.000(0.000)

__Iregion_13 -0.241**(0.066) -0.000(0.001) 0.000(0.000)

__Iregion_14 -0.380**(0.054) -0.007**(0.003) 0.002*(0.001)

__Iregion_15 -0.143**(0.068) -0.001(0.001) 0.000(0.000)

__Iregion_16 -0.173**(0.077) -0.012**(0.004) 0.002(0.001)

__Iregion_17 -0.195**(0.068) -0.000(0.002) 0.000(0.000)

__Iregion_18 -0.162**(0.056) -0.003(0.002) 0.001(0.000)

__Iregion_19 -0.003(0.104) -0.010**(0.004) 0.000(0.001)

__Iregion_20 -0.054(0.083) -0.000(0.002) 0.000(0.000)

__Iregion_21 -0.329**(0.082) 0.000(0.002) -0.000(0.001)

__Iregion_22 0.166**(0.051) -0.002*(0.001) -0.000*(0.000)

__Iregion_23 -0.169*(0.093) -0.002**(0.001) 0.000(0.000)

__Iregion_24 -0.357**(0.068) -0.004*(0.002) 0.001(0.001)

__Iregion_25 -0.113**(0.057) -0.006**(0.002) 0.001(0.000)

__Iregion_26 0.324**(0.044) -0.000(0.000) -0.000(0.000)

__Iregion_27 0.397**(0.046) 0.000*(0.000) 0.000(0.000)

__Iregion_28 0.789**(0.026) -0.000(0.000) -0.000(0.000)

__Iregion_29 0.232**(0.062) -0.001**(0.000) -0.000**(0.000)

__Iregion_30 0.240**(0.070) -0.001**(0.000) -0.000**(0.000)

__Iurban_2 0.558**(0.030) 0.011(0.007) 0.006(0.004)

__Ieducation_2 -0.021**(0.008) 0.027**(0.010) -0.001*(0.000)

__Ieducation_3 0.490**(0.016) 0.011**(0.003) 0.005**(0.002)

__Ieducation_4 0.884**(0.023) 0.001*(0.001) 0.001*(0.001)

__Iemployment_2 -0.027**(0.005) 0.012(0.019) -0.000(0.001)

__Iquintile_2 -0.367**(0.025) 0.008**(0.004) -0.003**(0.001)

__Iquintile_3 0.025(0.026) 0.008**(0.004) 0.000(0.000)

__Iquintile_4 0.415**(0.021) 0.012**(0.006) 0.005**(0.002)

__Iquintile_5 0.807**(0.010) 0.020**(0.006) 0.016**(0.005)

Residual 0.006**(0.002)

**Total 0.059**(0.005)**

Region 0.013**(0.003)

Urban residence 0.006(0.004)

Highest education level 0.006**(0.002)

Employment status -0.000(0.001)

Wealth quintiles 0.018**(0.006)

------------------------------------------------------------------------------------------

Observations 7,019

------------------------------------------------------------------------------------------

Standard errors in parentheses

* p<0.1, ** p<0.05

*Note*: __Iregion1 = Dodoma; __Iregion2 = Arusha; __Iregion3 = Kilimanjaro; __Iregion4 = Tanga; __Iregion5 = Morogoro; __Iregion6 = Pwani; __Iregion7 = Dar es Salaam; __Iregion8 = Lindi; __Iregion9 = Mtwara; __Iregion10 = Ruvuma; __Iregion11 = Iringa; __Iregion12 = Mbeya; __Iregion13 = Singida; __Iregion14 = Tabora; __Iregion15 = Rukwa; __Iregion16 = Kigoma; __Iregion17 = Shinyanga; __Iregion18 = Kagera; __Iregion19 = Mwanza; __Iregion20 = Mara; __Iregion21 = Manyara; __Iregion22 = Njombe; __Iregion23 = Katavi; __Iregion24 = Simiyu; __Iregion25 = Geita; __Iregion26 = Zanzibar north; __Iregion27 = Zanzibar south; __Iregion28 = Town west; __Iregion29 = Pemba north; __Iregion30 = Pemba south

__Ieducation_1 = no formal education; __Ieducation_2 = Primary education; __Ieducation_3 = Secondary education; __Ieducation_4 = Tertiary education

__Iurban_1 = urban location; __Iurban_2 = rural location

__Iemployment_1 = Not employed; __Iemployment_2 = employed

__Iquintile_1 = Q1(poorest quintile); __Iquintile_2 = Q2; __Iquintile_3 = Q3; __Iquintile_4 = Q4; __Iquintile_5 = Q5(richest quintile)

**Table A.11: Zambia**

------------------------------------------------------------------------------------------

Conc. Index Elasticity Contribution

------------------------------------------------------------------------------------------

Age 0.005**(0.002) 0.094**(0.038) 0.000*(0.000)

No. of children born -0.075**(0.005) -0.043**(0.015) 0.003**(0.001)

Household head -0.142**(0.022) -0.001(0.002) 0.000(0.000)

__Iregion_2 0.430**(0.032) -0.002(0.003) -0.001(0.001)

__Iregion_3 -0.314**(0.026) 0.003(0.003) -0.001(0.001)

__Iregion_4 -0.285**(0.034) 0.000(0.002) -0.000(0.001)

__Iregion_5 0.564**(0.020) -0.004(0.004) -0.003(0.002)

__Iregion_6 -0.348**(0.037) 0.002(0.002) -0.001(0.001)

__Iregion_7 -0.364**(0.041) 0.004(0.003) -0.002(0.001)

__Iregion_8 -0.165**(0.056) 0.000(0.002) -0.000(0.000)

__Iregion_9 0.033(0.053) 0.004(0.004) 0.000(0.000)

__Iregion_10 -0.413**(0.037) -0.001(0.002) 0.000(0.001)

__Iurban_2 0.538**(0.017) -0.012**(0.006) -0.007**(0.003)

__Ieducation_2 -0.212**(0.011) 0.022**(0.009) -0.005**(0.002)

__Ieducation_3 0.281**(0.012) 0.019**(0.008) 0.005**(0.002)

__Ieducation_4 0.855**(0.011) 0.007**(0.002) 0.006**(0.001)

__Iemployment_2 -0.001(0.010) 0.019**(0.005) -0.000(0.000)

__Iquintile_2 -0.336**(0.022) 0.000(0.003) -0.000(0.001)

__Iquintile_3 0.061**(0.026) -0.000(0.003) -0.000(0.000)

__Iquintile_4 0.452**(0.021) -0.002(0.004) -0.001(0.002)

__Iquintile_5 0.827**(0.011) 0.004(0.004) 0.004(0.004)

Residual 0.003**(0.001)

**Total 0.003(0.004)**

Region -0.006**(0.002)

Urban residence -0.007**(0.003)

Highest education level 0.007**(0.002)

Employment status -0.000(0.000)

Wealth quintiles 0.003(0.005)

------------------------------------------------------------------------------------------

Observations 7,302

------------------------------------------------------------------------------------------

Standard errors in parentheses

* p<0.1, ** p<0.05

*Note*: __Iregion1 = Central; __Iregion2 = Copperbelt; __Iregion3 = Eastern; __Iregion4 = Luapula; __Iregion5 = Lusaka; __Iregion6 = Muchinga; __Iregion7 = Northern; __Iregion8 = North western; __Iregion9 = Southern; __Iregion10 = Western

__Ieducation_1 = no formal education; __Ieducation_2 = Primary education; __Ieducation_3 = Secondary education; __Ieducation_4 = Tertiary education

__Iurban_1 = urban location; __Iurban_2 = rural location

__Iemployment_1 = Not employed; __Iemployment_2 = employed

__Iquintile_1 = Q1(poorest quintile); __Iquintile_2 = Q2; __Iquintile_3 = Q3; __Iquintile_4 = Q4; __Iquintile_5 = Q5(richest quintile)

**Table A.12: Zimbabwe**

------------------------------------------------------------------------------------------

Conc. Index Elasticity Contribution

------------------------------------------------------------------------------------------

Age 0.006**(0.002) 0.623**(0.073) 0.004**(0.002)

No. of children born -0.072**(0.006) -0.263**(0.035) 0.019**(0.003)

Household head -0.010(0.018) -0.004(0.006) 0.000(0.000)

__Iregion_2 -0.283**(0.038) 0.004(0.005) -0.001(0.001)

__Iregion_3 -0.065(0.043) 0.003(0.005) -0.000(0.000)

__Iregion_4 -0.079(0.058) 0.001(0.007) -0.000(0.001)

__Iregion_5 -0.375**(0.044) 0.001(0.002) -0.000(0.001)

__Iregion_6 -0.071*(0.039) 0.002(0.002) -0.000(0.000)

__Iregion_7 -0.074(0.053) -0.003(0.007) 0.000(0.001)

__Iregion_8 -0.225**(0.077) 0.002(0.005) -0.000(0.001)

__Iregion_9 0.601**(0.026) -0.009(0.008) -0.005(0.005)

__Iregion_10 0.698**(0.022) -0.003(0.002) -0.002(0.002)

__Iurban_2 0.643**(0.017) -0.015(0.010) -0.009(0.006)

__Ieducation_2 -0.348**(0.015) -0.045(0.027) 0.016*(0.009)

__Ieducation_3 0.117**(0.012) -0.082(0.055) -0.010(0.007)

__Ieducation_4 0.736**(0.018) 0.004(0.004) 0.003(0.003)

__Iemployment_2 0.120**(0.011) -0.002(0.011) -0.000(0.001)

__Iquintile_2 -0.375**(0.028) 0.004(0.006) -0.002(0.002)

__Iquintile_3 -0.011(0.030) 0.011**(0.005) -0.000(0.000)

__Iquintile_4 0.399**(0.023) 0.014(0.009) 0.006(0.004)

__Iquintile_5 0.818**(0.012) 0.038**(0.007) 0.031**(0.066)

Residual 0.007**(0.003)

**Total 0.055**(0.007)**

Region -0.010**(0.004)

Urban residence -0.009(0.006)

Highest education level 0.009**(0.003)

Employment status -0.000(0.001)

Wealth quintiles 0.035**(0.008)

------------------------------------------------------------------------------------------

Observations 4,805

------------------------------------------------------------------------------------------

Standard errors in parentheses

* p<0.1, ** p<0.05

*Note*: __Iregion1 = Manicaland; __Iregion2 = Mashonaland central; __Iregion3 = Mashonaland east; __Iregion4 = Mashonaland west; __Iregion5 = Matabeleland north; __Iregion6 = Matabeleland south; __Iregion7 = Midlands; __Iregion8 = Masvingo; __Iregion9 = Harare; __Iregion10 = Bulawayo

__Ieducation_1 = no formal education; __Ieducation_2 = Primary education; __Ieducation_3 = Secondary education; __Ieducation_4 = Tertiary education

__Iurban_1 = urban location; __Iurban_2 = rural location

__Iemployment_1 = Not employed; __Iemployment_2 = employed

__Iquintile_1 = Q1(poorest quintile); __Iquintile_2 = Q2; __Iquintile_3 = Q3; __Iquintile_4 = Q4; __Iquintile_5 = Q5(richest quintile)
